# Correlation Between Dietary Nutrition and Glymphatic System Activity in Healthy Participants

**DOI:** 10.7759/cureus.77860

**Published:** 2025-01-22

**Authors:** Miho Ota, Kiyotaka Nemoto, Hiroaki Hori, Ikki Ishida, Shinji Sato, Takashi Asada, Hiroshi Kunugi, Tetsuaki Arai

**Affiliations:** 1 Department of Psychiatry, Division of Clinical Medicine, Institute of Medicine, University of Tsukuba, Tsukuba, JPN; 2 Department of Mental Disorder Research, National Institute of Neuroscience, National Center of Neurology and Psychiatry, Tokyo, JPN; 3 Department of Behavioral Medicine, National Institute of Mental Health, National Center of Neurology and Psychiatry, Tokyo, JPN; 4 Department of Psychiatry, Teikyo University School of Medicine, Tokyo, JPN; 5 Department of Psychiatry, Division of Clinical Medicine, Institute of Medicine, University of Tsukuba, Ibaraki, JPN; 6 Section of Psychiatry and Behavioral Sciences, Tokyo Medical and Dental University Graduate School, Tokyo, JPN; 7 Department of Psychiatry, Memory Clinic Ochanomizu, Tokyo, JPN

**Keywords:** brain clearance system, diffusion tensor image, glymphatic system, oxidative stress, zinc

## Abstract

Background

Dietary nutrition is an important approach to the prevention and treatment of the physical and mental states of humans. Nowadays, many studies show the neuroprotective and antioxidative effects of nutrients, such as B vitamins, zinc, and iron, on the central nervous system (CNS). However, there were no studies focusing on the relationships between the serum concentration of nutrients and the brain glymphatic system activity and macroscopic waste clearance system, including reactive oxygen species.

Objectives

This study tries to evaluate the relationships between them using diffusion tensor image analysis along the perivascular space (DTI‑ALPS) index as the proxy of glymphatic system activity.

Methods

The subjects were 159 healthy participants who underwent 1.5-Tesla DTI (diffusion tensor imaging) and blood sampling. We computed the DTI‑ALPS index and estimated the relationships between the DTI‑ALPS index and the serum concentrations of vitamin B6, vitamin B12, folate, zinc, ferritin, and iron.

Results

There were significant positive correlations of the DTI‑ALPS index with age and sex. Additionally, we found that age, sex, and serum zinc level were good independent variables that predicted the dependent variable, the DTI‑ALPS index, as revealed by multiple regression analyses.

Conclusion

We found a significant correlation between brain clearance system activity and serum zinc levels in healthy participants. Though zinc is known to play an important physiological role in the CNS, excessive zinc accumulation or zinc deficiency might induce neurodegeneration. Further works with varying serum zinc concentrations would reveal the neuroprotective effect of zinc in its proper concentration.

## Introduction

Accumulating evidence has suggested the important role of dietary nutrients on physical and brain functioning. The possible roles of vitamins and minerals in the central nervous system (CNS) have been well-studied. For example, zinc plays an important role in the process of neurogenesis [1], and serum zinc levels in patients with Alzheimer’s disease (AD) and those with Parkinson’s disease were found to be significantly lower as compared with those of healthy controls [2]. Further, the imbalance in the metal levels, such as copper and iron, in the brains of AD patients has also been identified [3-5]. In addition, the B vitamins, particularly vitamin B6, vitamin B12, and folate, are suggested to be protective against AD and age-related cognitive decline through the suppression of homocysteine levels that increase oxidative stress [6-8].

Previous studies showed that the glymphatic system is involved in the movement of cerebrospinal fluid (CSF) and the clearance of harmful products, such as amyloid-beta, tau, and reactive oxygen species (ROS), and controls ROS-related inflammation [9-10]. Alternatively, oxidative stress and proinflammatory mediators can induce blood-brain barrier disruptions and may contribute to abnormal CSF-ISF (interstitial fluid) circulations, which might lead to glymphatic system dysfunction [11]. Nowadays, diffusion tensor imaging analysis along the perivascular space (DTI-ALPS), computed by DTI data, was noted as a marker for evaluating the glymphatic system activity with high reproducibility [12-13]. Previous studies using positron emission tomography detected significant correlations between the DTI-ALPS index and tau-deposition in patients with AD [14-15]. Significant correlations between the DTI-ALPS index and cognitive scores of AD have also been reported [12,14-15]. Yet, there was no DTI-ALPS study focusing on the effects of nutrients.

In this study, we examined the relationships between nutrients and the waste clearance system activity estimated by DTI-ALPS. We hypothesized that nutrients, such as B vitamins and zinc, might show neuroprotective effects in their proper concentration.

## Materials and methods

Participants

The study population consisted of healthy participants who were living in the earthquake-affected area in 2011 in Kitaibaraki, Japan. This research was conducted from 2011 to 2012, and we targeted 1019 residents aged ≥ 20 years old, of whom 190 agreed to undertake the MRI scan and blood sampling. All participants were rated with the Mini-Mental State Examination (MMSE). Those individuals who demonstrated a history of psychiatric illness or cognitive impairment were excluded from the study. Twenty-one individuals had previous history of mental illness, MMSE scores of 5 individuals were under 23, 2 individuals showed abnormal scores in the blood sampling, and 2 people contained artifacts in the MRI scan. As a consequence, 159 healthy subjects (34 male and 125 female) were finally involved in the study. We held a cross-sectional study to evaluate the relationships between glymphatic system activity and nutritional indices.

Ethics statement

We obtained written informed consent from each of the participants in this study after explaining the study. Ethical approval for this study was obtained from the ethics committee of the National Center of Neurology and Psychiatry, Japan (B2022-043).

Measurement of serum nutrients

Non-fasting venous blood samples were taken between 9:00 am and 3:00 pm from each participant to measure the concentration of serum vitamin B6, vitamin B12, folate, zinc, ferritin, and iron. These samples were collected in tubes and were centrifuged for 10 minutes at 3000 rpm. Serum concentrations were measured at SRL Inc. (Tokyo, Japan).

MRI data acquisition and diffusion tensor imaging along the perivascular space (DTI-ALPS) calculation

Brain MR studies were performed on a Philips MR system at 1.5-Tesla (Philips Medical Systems, Best, the Netherlands). DTI was performed in the axial plane (TE / TR, 66.75 / 8818 ms; FOV, 224 × 224 mm^2^; matrix: 128 × 128, 65 slices with thickness of 2 mm and no interslice gap, number of acquisition: 1). DTI images were gathered in 15 gradient directions with sensitivity of b= 0 and 800 s/mm^2^. We calculated the DTI-ALPS index by using an automated method previously described in detail [12,14]. We averaged the bilateral DTI-ALPS indices to step down the multiple comparison test.

Statistical analysis

The differences in clinical characteristics of the participants were estimated using two-sample t-tests. A *p-*value < 0.05 was regarded as statistically significant, and we used the Bonferroni correction for a multiple comparison test. As for the correlation analyses, we evaluated the relationships with the DTI-ALPS index and serum concentrations of nutrients by Pearson's correlation analysis. Further, simultaneous multiple regression analyses were carried out with the DTI-ALPS index as the dependent variable and clinical features as independent variables. A p-value of < 0.05 was regarded as statistically significant. Statistical analyses were performed using SPSS Statistics for Windows 23.0 software (IBM Japan, Tokyo, Japan).

## Results

**Figure 1:**
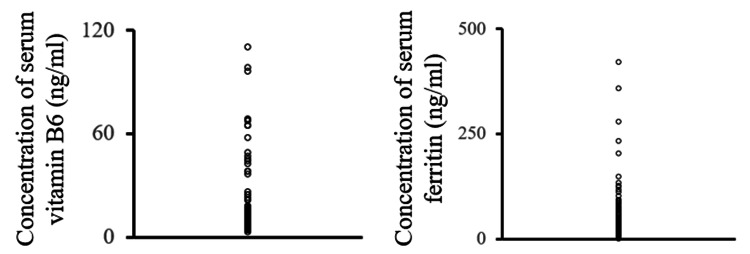
Serum concentration of vitamin B6 and ferritin in female participants Vitamin B6 and ferritin concentrations in female participants had a higher standard deviation than the mean value.

The clinical characteristics of the participants are shown in Table 1. There were significant differences in the DTI-ALPS index and serum ferritin concentration between the male and female healthy participants. The data of vitamin B6 and ferritin concentrations in females, having a higher standard deviation than the mean value, are plotted in Figure 1.

**Table 1:** Clinical characteristics of the participants ^*^p < 0.05: significant MMSE: Mini-Mental State Examination; DTI-ALPS: diffusion tensor imaging analysis along the perivascular space

	Male (N = 34)	Female (N = 125)	p-value
Age (years)	58.0 ± 15.0	54.8 ± 12.4	0.204
MMSE	28.1 ± 1.8	28.9 ± 1.7	0.014
Bilateral DTI-ALPS	1.4 ± 0.2	1.5 ± 0.2	0.002^*^
Vitamin B6 (pyridoxal, ng/ml)	14.5 ± 9.0	17.5 ± 19.7	0.192
Vitamin B12 (pg/ml)	375.4 ± 157.0	471.0 ± 361.7	0.135
Folate (ng/ml)	6.4 ± 2.3	7.3 ± 4.2	0.281
Ferritin (ng/ml)	110.9 ± 100.7	50.3 ± 62.6	0.002^*^
Zinc (µg/dl)	82.8 ± 23.7	77.4 ± 25.8	0.275
Iron (µg/dl)	95.8 ± 37.2	80.6 ± 35.5	0.030

There were significant correlations of the DTI-ALPS index with age and sex. However, we did not find any significant correlation of the DTI-ALPS index with serum concentrations of vitamin B6, B12, folate, iron, ferritin, or zinc (Table 2).

**Table 2:** Pearson's correlation analyses with bilateral DTI-ALPS ^*^p < 0.05: significant DTI-ALPS: diffusion tensor imaging analysis along the perivascular space

	Correlation coefficient	p-value
Sex	0.25	0.002^*^
Age	-0.35	< 0.001^*^
Vitamin B6 (pyridoxal, ng/ml)	-0.03	0.732
Vitamin B12 (pg/ml)	0.01	0.878
Folate (ng/ml)	-0.06	0.491
Ferritin (ng/ml)	-0.08	0.290
Zinc (µg/dl)	0.13	0.103
Iron (µg/dl)	-0.09	0.253

**Figure 2:**
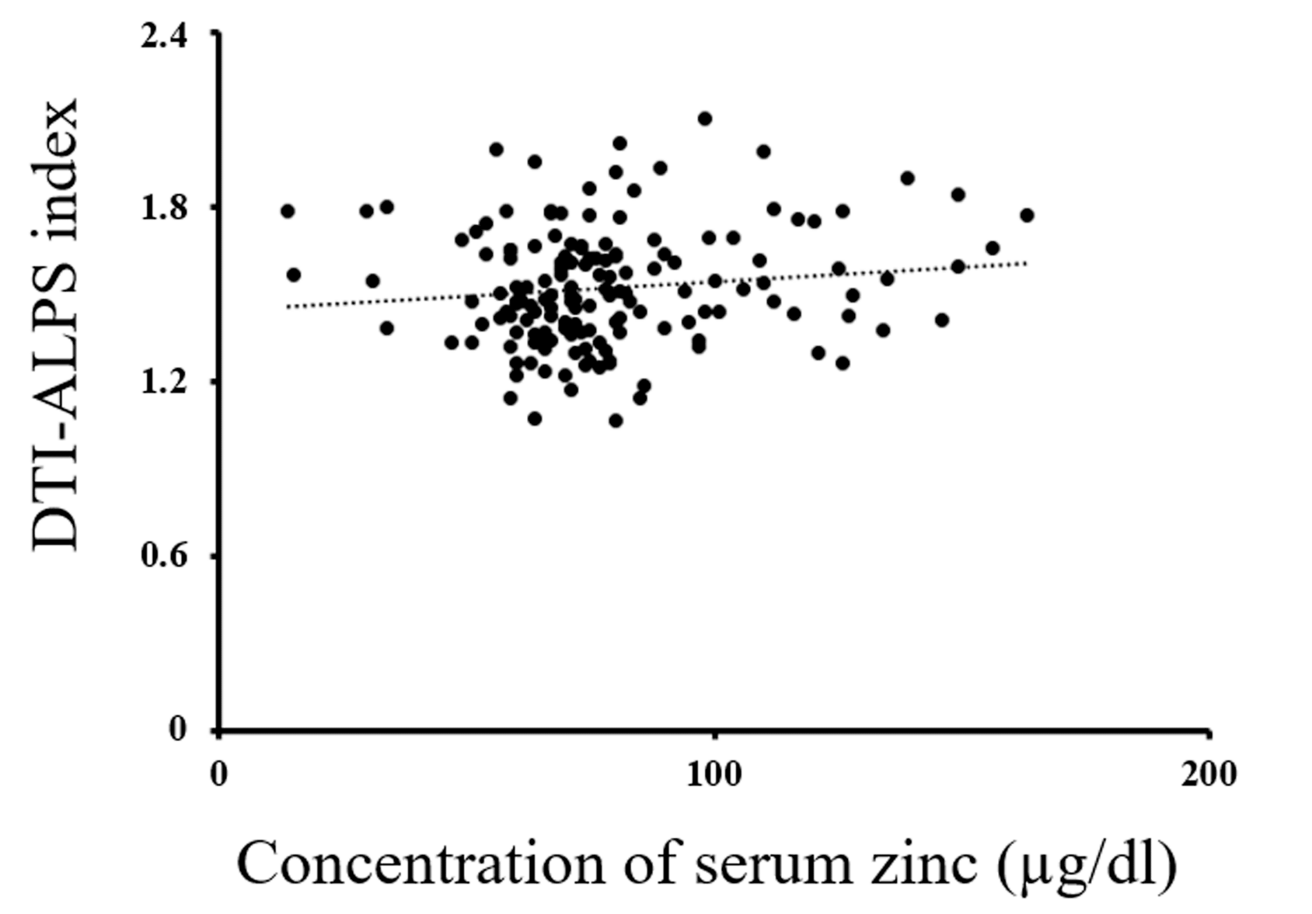
Relationship between the serum zinc concentration and DTI-ALPS index The scatter plot of the serum zinc concentration and the DTI-ALPS index in healthy participants is shown. The dotted line is the regression line. DTI-ALPS: diffusion tensor imaging analysis along the perivascular space

When we calculated the simultaneous multiple regression analysis to identify the variables that were associated with DTI-ALPS, we found that the combination of age (β = -0.34, p-value < 0.001), sex (β = 0.23, p-value = 0.002), and serum zinc concentration (β = 0.17, p-value = 0.020) were significantly correlated with the DTI-ALPS index (Figure 2, Table 3). The other nutrients did not reach statistical significance.

**Table 3:** Results of the multiple regression model ^*^p < 0.05: significant

Independent variable	Standardized coefficient (beta)	t value	p-value
(Constant)		14.40	< 0.001^*^
Age	-0.34	-4.70	< 0.001^*^
Sex	0.23	3.14	0.002^*^
Zinc (µg/dl)	0.17	2.34	0.02^*^
Vitamin B6 (pyridoxal, ng/ml)	-0.03	-0.40	0.688
Vitamin B12 (pg/ml)	0.06	0.76	0.449
Folate (ng/ml)	-0.01	-0.12	0.908
Ferritin (ng/ml)	0.06	0.76	0.446
Iron (µg/dl)	0.01	0.09	0.926

## Discussion

We found significant relationships between the DTI-ALPS index and age and gender. Additionally, there was a significant positive correlation between the DTI-ALPS index and serum zinc concentration, controlling for age and sex. In other words, participants with a high serum zinc concentration showed high glymphatic system activity. To our knowledge, this is the first study that evaluated the relationships between glymphatic system activity and dietary nutrients. One of the strengths of this report was that it was a rare study in which a large number of participants across a wide age range underwent nutritional investigations and MRI examinations.

Some previous studies showed that the DTI-ALPS indices were negatively correlated with age and sex [16-17], and these points were congruent with our study. It is known that zinc has an important role in cognition, regulating mood, and performs a neuroprotective function as an antioxidant [18-19]. Further, serum zinc levels in patients with AD were significantly lower as compared with controls [2]. Regarding the antioxidative function, zinc exhibits antioxidant properties via several molecules and enzymes, such as the catalysis of copper/zinc-superoxide dismutase, the protection of the protein sulfhydryl group, and the upregulation of metallothionein (MT) expression, which exhibits antioxidant functions [20]. The glymphatic system modulates the clearance of ROS and controls ROS-related inflammation [9-10]; and vice versa, oxidative stress also leads to glymphatic system dysfunction [11]. This antioxidant effect of zinc might support the maintenance of glymphatic system activity.

Iron and ferritin have also been suggested to affect mood and cognition through the treatment of iron deficiency anemia [21-22]. However, another study pointed out that a significant relationship between hemoglobin and cognitive function was detected in children with iron deficiency, whereas no similar evidence was found in iron-sufficient children [21]. Further, CSF ferritin level was strongly associated with CSF apolipoprotein E (APOE) levels and was elevated by the Alzheimer's risk allele, APOE-ɛ4 [23]. Other papers detected that CSF levels of ferritin were associated with prodromal disease progression of people with high β-amyloid pathology [5]. We did not find a relationship between the serum concentration of iron or ferritin and the DTI-ALPS index. It might have resulted from the fact that the relationship between plasma ferritin and CSF ferritin was modest [3]. Further study on CSF iron concentration might shed light on this topic.

On the other hand, some papers showed the effectiveness of B vitamins, such as vitamin B6, B12, and folate, on the CNS function through antioxidative functions and showed that supplementation of these factors prevented the onset of dementia, cognitive decline, and Alzheimer's disease [23-25]. However, the relationships between the B-vitamins concentration and the DTI-ALPS index did not reach statistical significance in this study. B vitamins possess a water-soluble property, and the excess of these vitamins is excreted in urine. This point might obscure our results of correlation analyses.

Limitation

First, the ratio of males in our participants was relatively low. It is well-known that the DTI-ALPS index of males is relatively low as compared to women [26], and women show a lower level of serum ferritin and iron than men. These points might affect the statistical results. Further trials with larger male participants would clarify these points. Next, vitamin B and minerals are influenced by diet, so in the future blood samples should be taken under single conditions. Based on the relationship with diet, it may be necessary to exclude lifestyle influences in the future.

## Conclusions

We found a significant correlation between the brain clearance system activity and serum zinc levels in healthy participants. In addition to dysgeusia, reduced blood levels of zinc may also be seen in sleep disorders, depressed mood, and cognitive dysfunction. It is also known that zinc exhibits antioxidant properties via several molecules and enzymes, and zinc might affect the glymphatic system as a protective agent by blocking oxidative stress. Glymphatic system dysfunction can lead to the accumulation of amyloid and tau, and zinc may be effective in preventing amyloid- and tau-related neurocognitive disorders such as AD and frontotemporal lobar degeneration.
